# The physical mechanism of magnetic field controlled magnetocaloric effect and magnetoresistance in bulk PrGa compound

**DOI:** 10.1038/srep14970

**Published:** 2015-10-12

**Authors:** X. Q. Zheng, H. Wu, J. Chen, B. Zhang, Y. Q. Li, F. X. Hu, J. R. Sun, Q. Z. Huang, B. G. Shen

**Affiliations:** 1 State Key Laboratory for Magnetism, Institute of Physics, Chinese Academy of Sciences, Beijing 100190, People’s Republic of China; 2 School of Materials Science and Engineering, University of Science & Technology of Beijing, 100083, People’s Republic of China; 3 NIST Center for Neutron Research, National Institute of Standards & Technology, Gaithersburg, MD 20899, USA; 4 Department of Materials Science and Engineering, University of Maryland, College Park, MD 20742-2115, USA; 5 Beijing Institute of Aerospace Testing Technology, China Aerospace Science & Technology Corporation, Beijing, 100074, People’s Republic of China

## Abstract

The PrGa compound shows excellent performance on the magnetocaloric effect (MCE) and magnetoresistance (MR). The physical mechanism of MCE and MR in PrGa compound was investigated and elaborated in detail on the basis of magnetic measurement, heat capacity measurement and neutron powder diffraction (NPD) experiment. New types of magnetic structure and magnetic transition are found. The results of the NPD along with the saturation magnetic moment (*M*_S_) and magnetic entropy (*S*_M_) indicate that the magnetic moments are randomly distributed within the equivalent conical surface in the ferromagnetic (FM) temperature range. PrGa compound undergoes an FM to FM transition and an FM to paramagnetic (PM) transition as temperature increases. The magnetizing process was discussed in detail and the physical mechanism of the magnetic field controlled magnetocaloric effect (MCE) and the magnetoresistance (MR) was studied. The formation of the plateau on MCE curve was explained and MR was calculated in detail on the basis of the magnetic structure and the analysis of the magnetizing process. The experimental results are in excellent agreement with the calculations. Finally, the expression of MR = *β*(T)X^2^ and its application conditions were discussed, where X is *M*(*H*)/*M*_eff_, and M_eff_ is the paramagnetic effective moment.

Ever since the discovery of giant magnetoresistance (GMR) in Fe/Cr multilayers^1^, much attention has been paid to artificial multilayer systems^2^. GMR effect can be observed when an antiferromagnetic (AFM) arrangement of the consecutive ferromagnetic (FM) layers has been forced into a complete FM alignment by an external magnetic field. The occurrence of GMR effects in multilayer systems is usually ascribed to the spin-dependent electron scattering both at layer boundaries and within each layer^3^,^4^. In addition to these artificial magnetic multilayers, many other systems such as granular alloys, oxides and bulk intermetallic compounds were also found to have significant magnetoresistance (MR) effects. Rare earth (*R*) based intermetallic compounds is one important category of bulk GMR materials, such as Ce(FeRu)_2_^5^, Gd_2_In^6^, Gd_5_(Si_2_Ge_2_)^7^ and PrGa^8^, and several models have been reported to describe MR^9^,^10^. Therefore, it would be very interesting to investigate the physical mechanism of MR from the relationship between electron transport and magnetic structure^6^.

Magnetic refrigeration based on magnetocaloric effect (MCE) is different from the conventional refrigeration based on gas compression/expansion. And magnetic refrigeration is considered to be a promising technology used for cooling in the future for its environmental safety and high efficiency^11^. People have done lots of works on studying the magnetic properties, MCE and MR of many materials including R based intermetallic compounds^12^,^13^,^14^. *R*Ga compounds show excellent performance on MR and MCE^15^,^16^,^17^. Early studies show that most of the RGa compounds exhibit a spin reorientation (SR) transition and an FM to paramagnetic (PM) transition as temperature increases^18^. However, no SR transition is observed in PrGa according to the previous Mössbauer spectroscopy experiment^18^. The magnetic property, MR and MCE of PrGa compound have been reported^8^,^19^, but the magnetic structure and physical mechanism of MR and MCE are still not clear.

PrGa was reported to be a ferromagnetic material and the Curie temperature was determined to be 32 K and 36 K in two different previous literatures^14^,^18^. In our previous work, we found that PrGa compound undergoes two sequential magnetic transitions with increasing temperature: an FM to antiferromagnetic (AFM) transition at *T*_t_ ~ 27 K and an AFM to PM transition at *T*_C_ ~ 37 K, respectively^8^. It should be noted that the AFM ground state between *T*_t_ and *T*_C_ was judged by the isothermal magnetization (*M-H*) curve only. We also studied the thermal expansion, MCE and MR of PrGa compounds in details^8^. Our previous studies showed that a nearly constant value of ΔS_M_ was observed when the field changes from 0 to 5 T^19^. A nearly constant ΔS_M_ has also been observed in LaFe_11.44_Al_1.56_ compound^20^, while the cause of plateau on the MCE curve in PrGa compound is different from that in LaFe_11.44_Al_1.56_ compound. The physical mechanism of plateau-type MCE in PrGa compound need to be studied further. In addition the MR of PrGa compound around 30 K is as high as 35%, which is greater than or comparable to that of most other R based intermetallic compounds such as Ce(FeRu)_2_^5^, Gd_2_In^6^ and Gd_5_(Si_2_Ge_2_)^7^. Although the magnetoresistance in granular ferromagnetic system and the doped magnetic semiconductors has been studied^9^,^10^, the magnetic field controlled physical mechanism of MR in bulk materials is still lacking. Therefore, it is necessary to study the physical mechanisms of MCE and MR in bulk PrGa compound.

In this work, we studied the magnetic structure of PrGa compound by means of magnetic measurement, heat capacity measurement and high-resolution neutron powder diffraction (NPD) experiments. Then the magnetic transition and magnetizing process were described in detail. The origin of the plateau on MCE curve was explained. A physical model was built to describe the mechanism of electron-magnon scattering in PrGa compound.

## Results

### Saturation moment (M_S_) and Magnetic entropy (*S*_M_)

**Figure 1:**
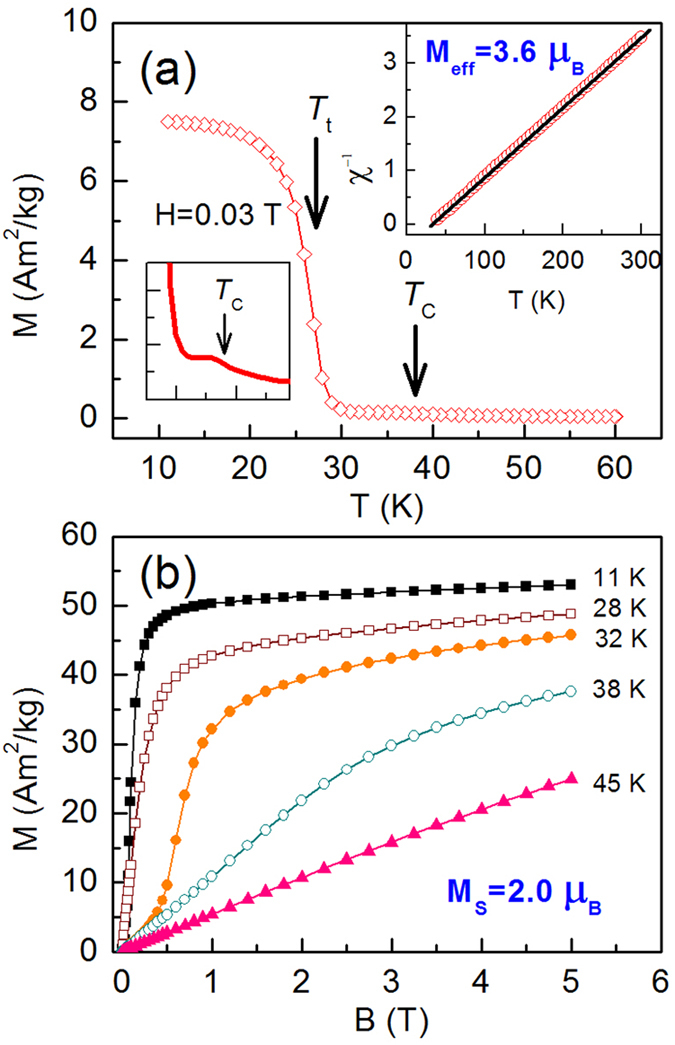
Basic magnetic property. (**a**) Thermal magnetization curve of PrGa compound in a magnetic field of 0.03 T. The inset is the temperature dependence of the magnetic susceptibility inverse. (**b**) Isothermal magnetization curve of PrGa compound at 11 K, 28 K, 32 K, 38 K and 45 K, respectively.

NPD measurement indicated that the as-synthesized polycrystalline PrGa sample is single phase. The thermal magnetization curve at H = 0.03 T is shown in Fig. 1(a) and the temperature dependence of χ^−1^ up to 300 K is shown in the inset of Fig. 1(a), where χ is the magnetic susceptibility. Our previous result of the thermal magnetization curve indicated that PrGa compound undergoes two magnetic transitions as temperature changes^8^,^19^. These two magnetic transitions will be discussed in detail in the following section. The linear relationship of χ^−1^ and temperature in the high temperature range indicates that the Curie-Weiss law is appropriate here. According to the Curie-Weiss law and the definition of *χ*, *M*_*eff*_ is calculated to be 3.6 *μ*_*B*_ from the slope of the linear fitting curve. Isothermal magnetization curves at various temperatures have been measured and reported^19^. Here the curves at 11 K, 28 K, 32 K, 38 K and 45 K were quoted and plotted in Fig. 1(b), respectively. The MH curve at 5 K (not shown in Fig. 1) has also been measured and it is almost coincided with the MH curve at 11 K in high field range. For the isothermal magnetization curve at 5 K and 11 K, the magnetization increases with field increasing and reaches its saturation value rapidly, indicating that it is reasonable to calculate saturated magnetization (M_S_) from the MH curve at 5 K or 11 K. M_S_’s at these two temperature are calculated to be the same value of 2.0 *μ*_*B*_, which is the average value per Pr atom. The value of *M*_*S*_ is considerably smaller than that of *M*_*eff*_. As a matter of fact, *M*_*S*_ is the macroscopic FM-ordered component of the magnetic moments. However, *M*_*eff*_ is the real magnetic moment of every Pr atom. The great difference between *M*_S_ and *M*_*eff*_ indicates that the directions of the magnetic moments in PrGa are not completely uniform below 11 K.

**Figure 2:**
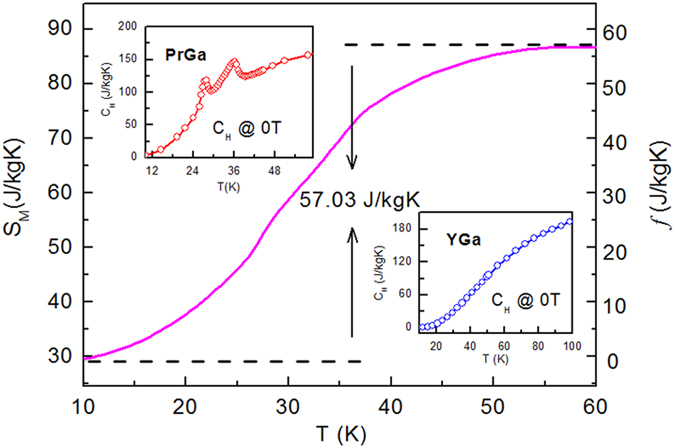
The entropy contributed from magnetic moments. Temperature dependence of the magnetic entropy of PrGa compound. The two insets are the heat capacity for PrGa and YGa, respectively.

In order to study the magnetic ground state at low temperature, we investigated the degree of magnetic order, which is known as magnetic entropy (*S*_*M*_). The total entropy can be calculated from the integration of the heat capacity data. The heat capacity data at zero field for PrGa and YGa was measured and shown in the inset of Fig. 2. The total entropies (S_tot_) of PrGa and YGa compound were calculated with *T* = 10 K as reference temperature. The S_tot_ mainly contains electronic contribution, lattice contribution and magnetic contribution. The non-magnetic contributions of S_tot_ can be considered to be the same in PrGa and YGa compounds because they show the same crystal structure. Then *S*_*M*_ of PrGa was obtained by subtracting *S*_tot_ of YGa from *S*_tot_ of PrGa. The above calculation method has been reported in literature^21^,^22^. Now we introduce a new function *f* (T).


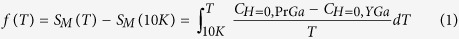


The right side of eq. (1) can be calculated and the curve was plotted in Fig. 2. *S*_*M*_ increases as temperature goes up and it changes more rapidly in the temperature range between *T*_t_ and *T*_C_ than in other temperature ranges. When the temperature exceeds *T*_C_, *S*_M_ changes slowly and reaches its maximum value gradually.


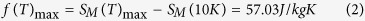


*S*_*M*_(*T*)_max_ or *S*_*M*_ in PM zone can be calculated from the total angular quantum number^23^. Then we can obtain 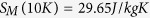. PrGa compound is in stable FM ordered state and no magnetic transition is observed below 11 K. So the large value of *S*_M_ at 10 K is unusual. This indicates that the magnetic moments of PrGa are not parallel completely at 10 K, and more complex magnetic structure may exist. This conclusion is in accord with the analysis of *M*_*S*_ and *M*_*eff*_.

### Magnetic structure, magnetic transition and magnetizing process

**Figure 3:**
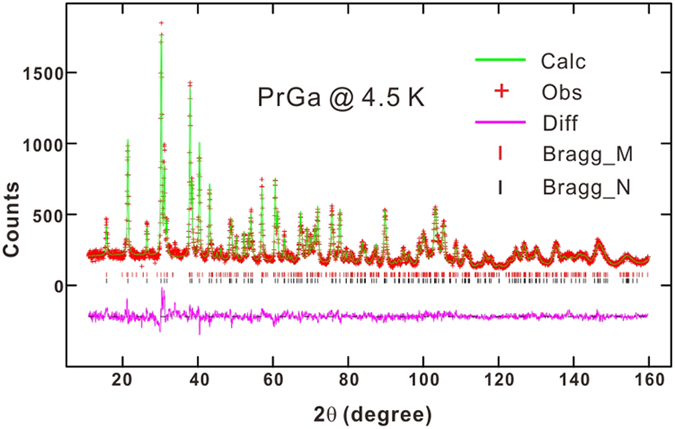
The neutron powder diffraction pattern of PrGa at 4.5 K. The observed data and the calculated patterns are plotted in cross and solid line, respectively. The solid line at the bottom is the difference between the observed and calculated data. The Bragg positions for the nuclear and magnetic structure are marked as short vertical lines.

**Figure 4:**
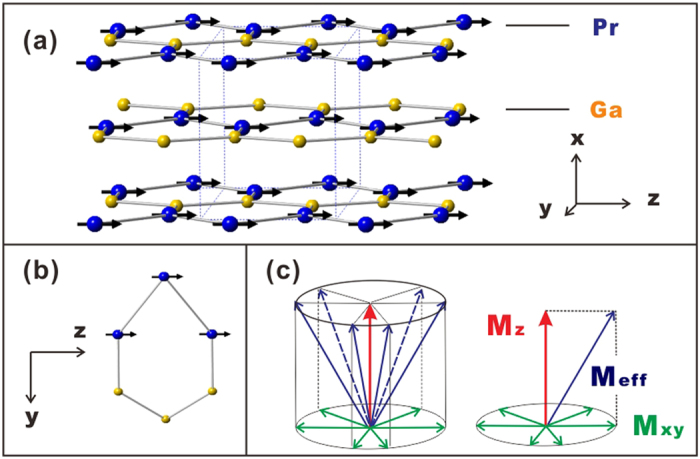
Crystal and magnetic structure. (**a**) Distribution of magnetic moments in each unit cell. (**b**) The distribution of magnetic moments of the hexagon ring in yz crystal plane. (**c**) The cone-surface distribution of the actual magnetic moments in PrGa and the vector decomposition of the magnetic moments.

Both *M*_S_ and *S*_M_ results indicate that complex magnetic structure exists in PrGa compound at low temperature. High resolution NPD experiment was then employed to study the magnetic structure of PrGa. The experiments are performed at 4.5 K, 15 K, 20 K, 25 K, 28 K, 30 K, 33 K, 36 K, 40 K and 295 K, respectively. Fitting and calculations are carried out by Rietveld refinement method afterwards. The observed and the calculated NPD patterns at 4.5 K are shown in Fig. 3. Both the nuclear and the magnetic structure models are involved in the calculation. NPD results show that PrGa compound has a pure phase and it crystalizes in a *CrB*-type orthorhombic structure (space group #63 *cmcm*). All of the atoms distribute in a form of layers perpendicular to *a* axis, and atoms are connected in a form of hexagon rings within each layer. The crystal structure of PrGa compound is almost the same as other RGa compounds, except for the small differences in the lattice constant^18^. The detailed crystal structure of PrGa compound is shown in Fig. 4(a,b) and the detailed parameters of crystal structure are listed in Table 1. The magnetic moments are FM-ordered along crystallographic *c* axis and the ordered magnetic moment is determined to be 2.6 *μ*_B_ at 4.5 K. The value of the ordered magnetic moment is calculated to be 2.54 *μ*_B_ and 2.5 *μ*_B_ by interpolation method at 5 K and 11 K, respectively. The deviation between M_S_ (2.0 *μ*_B_) and M_neu_ (2.54 *μ*_B_) at 5 K may be related to the different experimental setups. In the magnetic measurement, the sample is in the form of small granules, in which there are many unordered crystal boundaries and domain walls. In the neutron diffraction experiment, the effect of crystal boundaries or domain walls can be ignored approximately. Considering that M_S_ is much smaller than *M*_*eff*_ and *S*_M_ at 10 K is considerably larger than zero, we can assume that the magnetic moments point to different directions but with a fixed deviation angle from z axis and the value of 2.5 *μ*_B_ is the projection of the magnetic moments on z axis. If we draw all of the magnetic moments at the same atom site, we can see that they are distributed randomly within a conical surface. The sketch of the magnetic structure of PrGa is shown in Fig. 4(c). The real magnetic moment is *M*_*eff*_ and every magnetic moment can be resolved into two components. *M*_z_ and *M*_xy_ are the components along the z axis and within the xy crystal plane, respectively. Furthermore, *M*_z_ is long-range FM-ordered but M_xy_ is distributed randomly without any long-range order. *M*_*eff*_, *M*_z_ and *M*_xy_ have the following relation:


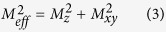


**Figure 5:**
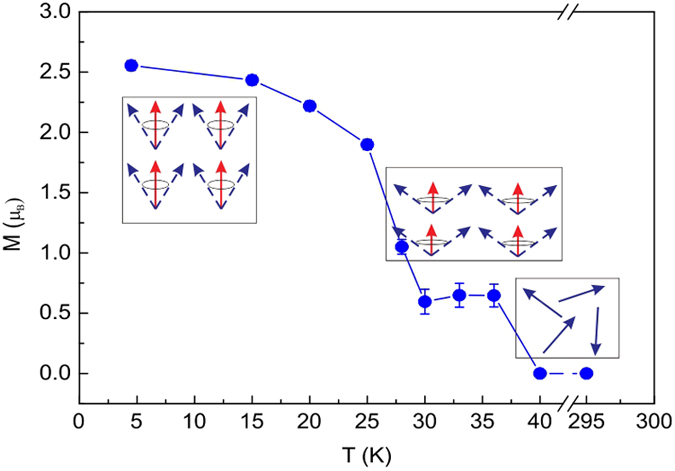
Evolution of magnetic structure as temperature changes. Temperature dependence of the ordered magnetic moment obtained from the powder neutron diffraction experiment. The ordered magnetic moments are along the crystallographic z-axis. M_z_ and M_eff_ are represented in red and blue arrows, respectively.

All the magnetic structures at different temperatures were solved out. The detailed results are listed in Table 1. The temperature dependence of the ordered magnetic moment and the sketch of the magnetic structures are shown in Fig. 5. As temperature increases, the magnetic moment shows a sudden decrease around *T*_t_ ~ 28 K, whereas the direction of the ordered magnetic moment remains the same. At *T*_C_ ~ 38 K the ordered magnetic moment decreases to zero, which indicates an FM to PM transition. Therefore we can conclude that PrGa compound undergoes an FM to FM magnetic transition at *T*_t_ and an FM to PM transition at *T*_C_ as temperature increases. According to the results of Mossbauer spectroscopy experiments, a magnetic spin reorientation transition from bc-plane toward the a-axis with decreasing temperature was observed for NdGa, SmGa, HoGa and ErGa compounds^18^. But for PrGa compound, there is no such reorientation of the ordered magnetic moments around T_t_, though the crystal structure of PrGa compound is almost the same as other RGa compounds. Furthermore, lattice parameters a, b and c change as temperature increases, but only c shows an obvious response to both the FM to FM transition and the FM to PM transition, because the change of magnetic structure at transition temperatures occurs mainly along the z axis.

**Figure 6:**
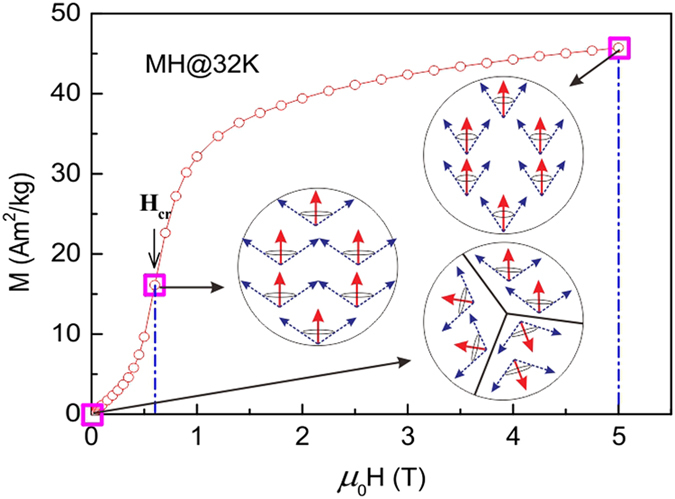
Evolution of magnetic structure as field changes. Isothermal magnetization curve of PrGa compound at 32 K and the corresponding magnetic structure in every magnetizing stage. M_z_ and M_eff_ are symbolized in red and blue arrows, respectively. Domain walls under zero field are represented by the black lines in the inset.

As we mentioned in our previous work^8^, the isothermal magnetization curves showed a typical AFM-like characteristics in the temperature range between *T*_t_ and *T*_C_. However, neutron diffraction results show that PrGa compound is an FM-ordered state in this temperature zone. We take the representative isothermal magnetization curve at 32 K to reinterpret the magnetizing process in PrGa compound. The isothermal magnetization curve at 32 K is quoted and the magnetic structure sketches in different field zones have been plotted in Fig. 6. The magnetizing process can be divided into two stages by the critical field (*H*_cr_), which is determined by the maximum of *d*M/*d*H. For PrGa compound, H_cr_ is the critical field where the metamagnetic transition begins and *H*_cr_ is calculated to be 0.6 T at 32 K. In the range of *H* ≤ *H*_cr_, there are many magnetic domains with different FM-ordered directions, so the macroscopic magnetization is zero at the beginning. The magnetization increases gradually with increasing magnetic field. In this stage, magnetizing process is mainly achieved by means of domain-wall displacement. Inside of every magnetic domain, the projection of every magnetic moment on z axis does not really change. Since the ordered magnetic moment is rather small at 32 K (see Fig. 5), the magnetization changes slowly in this stage. As discussed above, the *S*_M_ of PrGa compound also hardly changes in this stage. In the range of *H* > *H*_cr_, the domain-wall displacement reaches its limits. As magnetic field continues to increase, the magnetizing process is achieved by means of magnetic moment rotation. That is, the direction of every magnetic moment tends to align with z axis gradually. As a result, *S*_M_ of PrGa compound substantially changes in this stage. The most remarkable difference between the above two stages is that the magnetizing process is irrelevant to the degree of magnetic order in the first stage, but it is closely related in the second stage. This conclusion is important in interpreting the MCE and MR in PrGa compound in the following section.

### Magnetocaloric effect (MCE)

**Figure 7:**
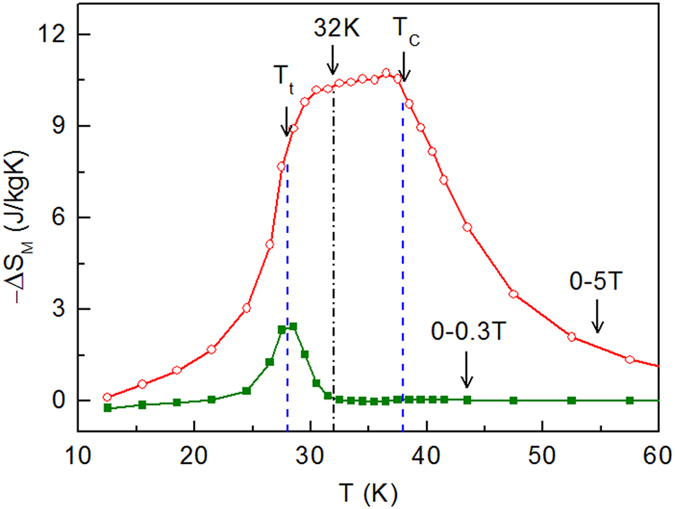
Magnetocaloric effect. Temperature dependence of –ΔS_M_ for a field change of 0–0.3 T and 0–5 T, respectively.

The MCE of PrGa has been studied in detail in our previous work^19^, where a plateau was observed on the magnetic entropy change (ΔS_M_) curve under a field change of 0–5 T. We redrew the ΔS_M_ curves (Fig. 7) calculated from the Maxwell relation for a field change of 0–0.3 T and 0–5 T, respectively. Figure 7 also shows that only for a large field change can we observe the plateau. When T < *T*_t_ or T > *T*_C_, the value of –Δ*S*_M_ is relatively small regardless of the field change. The temperature range between *T*_t_ and *T*_C_ is the most important zone for the formation of the plateau. Again we take 32 K data to interpret the cause of the plateau. When the field change is 0–0.3 T, the magnetizing process is in the first stage. As we have mentioned before, magnetizing process is achieved by domain-wall displacement, and S_M_ hardly changes in this stage. The value of –ΔS_M_ is almost zero and no plateau is observed for a field change of 0–0.3 T. When the magnetic field exceeds *H*_cr_, the magnetizing process steps into the second stage and all the magnetic moments begin to rotate toward z axis. Δ*S*_M_ changes significantly and thus a large ΔS_M_ value is observed for a field change of 0–5 T. In addition, the large value is also related to the lattice change around T_t_. It can be seen that the formation of a plateau is closely related to the magnetic structure, magnetic transition and magnetizing process in PrGa compound.

### Magnetoresistance (MR)

The magnetic structure, magnetic transition and magnetizing process also affect MR in PrGa compound. The electrical resistivity in magnetic materials includes residual resistivity (*ρ*_0_), resistivity due to electron-electron scattering (*ρ*_E_), resistivity due to electron-phonon scattering (*ρ*_L_) and resistivity due to the electron-magnon interactions (*ρ*_M_)^7^. Among them, magnetic field mainly affects the *ρ*_M_ term. *ρ*_M_ increases with increasing *S*_M_ and reaches to its maximum value when the temperature exceeds *T*_C_^24^. Since the FM-ordered magnetic component is along z axis, spin polarization of electrons exists. The component in the xy crystal plane is in the disordered state, therefore the electron motion within the xy plane is almost forbidden. The only motion path of electrons is thus along z axis, and the obstacle of the movement mainly comes from the disordered *M*_xy_. Therefore, we assume that the resistance caused by the magnetic moments is proportional to the projected area of the circular cone in the xy crystal plane (Fig. 4(c)). The above assumption can be expressed in the following formula:


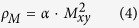


Coefficient α is a constant.

Since MR mainly comes from magnetic contribution in PrGa compound, at the fixed temperature of *T*, the change of electrical resistivity with different magnetic fields can be written as follows:


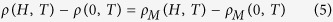


At zero field, the non-magnetic part of resistivity can be written as the multiple of the magnetic part, and then the total resistivity at zero field can be written as following:


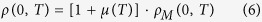


where *μ*(*T*) is a constant related to temperature. MR is usually written in the following formula:^24^,^25^


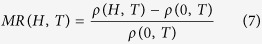


Put eq. (5) and (6) into eq. (7), we can get a new form of MR:


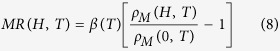


It should be noted that *μ*(*T*) has been replaced by *β*(*T*) on the basis of 
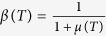
. Considering eq. (3), we can get the following expression:


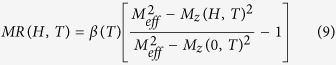


**Figure 8:**
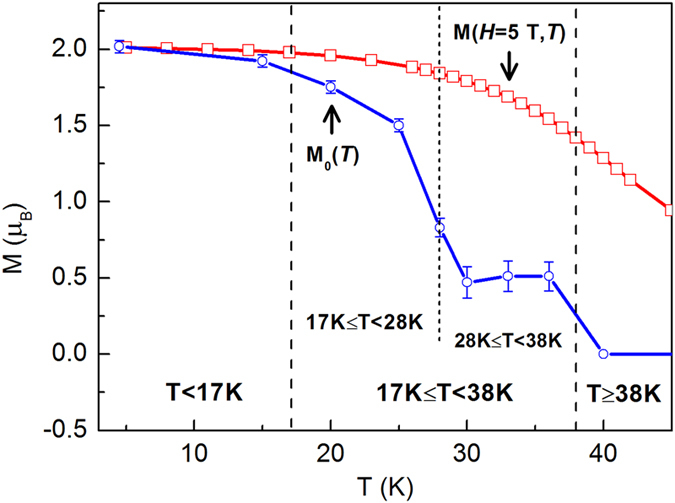
The divided temperature zones based on magnetic structure. The revised M_0_(T) curve and the thermal magnetization curve in a field of 5 T. The whole temperature range is divided into three zones and the boundary is marked as dotted lines.

Then it is the key question to determine M_z_(*H*, *T*) and M_z_(0, *T*). M_z_(0, *T*) is the FM-ordered component of magnetic moments at zero magnetic field and it cannot be detected from the macroscopic magnetic measurement because of the existence of magnetic domains. While M_z_(0, *T*) can be determined accurately by NPD results at zero field. The deviation of M_z_(0, *T*) obtained from bulk compound and powder sample has been discussed. In order to study MR in bulk PrGa compound, we revised the M_z_(0, *T*) obtained from NPD experiment by taking M_z_(0, 5 K) as a reference. The revised M_z_(0, *T*) is marked as M_0_(*T*) and it was plotted in Fig. 8. The expression of M_*z*_(*H*, *T*) is very complex, because it is not only temperature-dependent but also field-dependent. In order to discuss M_z_(*H*, *T*), the temperature dependence of magnetization at the field of 5 T (M(*H* = 5 T, *T*)) is also shown in Fig. 8. The whole temperature range is divided into three parts by *T* = 17 K and *T* = 38 K:

1) T < 17 K

The magnetizing process is generally realized by domain-wall displacement and magnetic moment rotation sequentially as magnetic field increases. M(*H* = 5 T, *T*) is a macroscopic physical quantity, and if M(*H* = 5 T, *T*) is no more than M_0_(*T*), we can conclude that the magnetizing process is only in the first stage and M_z_ does not really change even though the magnetic field is as high as 5 T. From Fig. 8 we can see that there is almost no difference between M_0_(*T*) and M(*H* = 5 T, *T*) in this temperature range. So, we have the following expression:


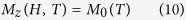


2) 17 K ≤ T < 38 K

In this temperature range, M(*H* = 5 T, *T*) is considerably larger than M_0_(*T*), which indicates that the magnetizing process experiences both stages as magnetic field increases from 0 to 5 T. Also it should be noted that *H*_cr_ is obtained in this temperature range. As we discussed on the magnetizing process at 32 K, *H*_cr_ is the field separatrix of the two magnetizing stages. And M(*H*, *T*) can be considered as the measure of M_z_(*H*, *T*), when the magnetic field exceeds *H*_cr_. So M_z_(*H*, *T*) can be expressed in the following piecewise function:


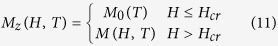


3) T≥38 K

In this temperature range, PrGa compound is in the PM state and every magnetic moment distributes randomly at zero field. When the magnetic field is applied, the macroscopic magnetization M(*H*, *T*) is the ordered component of the magnetic moment along z axis. So, we have the following expression:


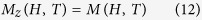


Putting eq. (10, 11, 12) into eq. (9), we can obtain MR(*H*, *T*). In order to simplify the expression of MR, we make the following variable transformation:


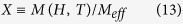



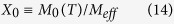



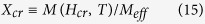


Then, MR can be expressed below:

1) T < 17 K


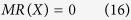


2) 17 K ≤ T < 38 K


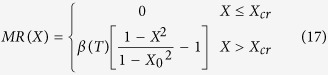


3) T ≥ 38 K


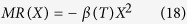


## Discussion

**Figure 9:**
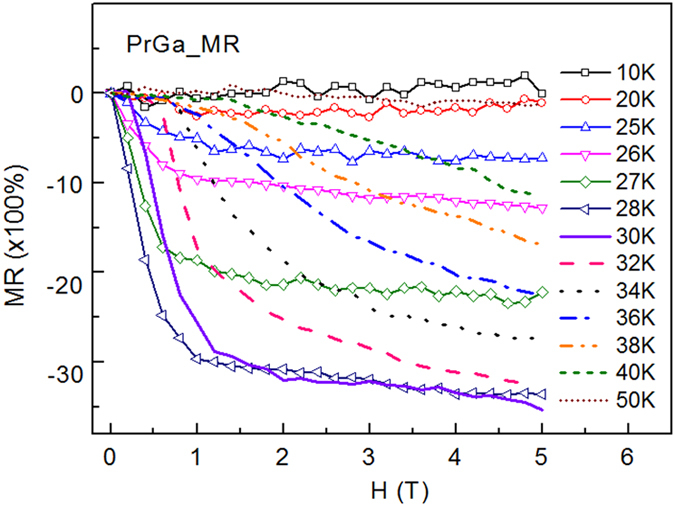
Magnetoresistance. Magnetic field dependence of magnetoresistance of PrGa compound at various temperatures.

**Figure 10:**
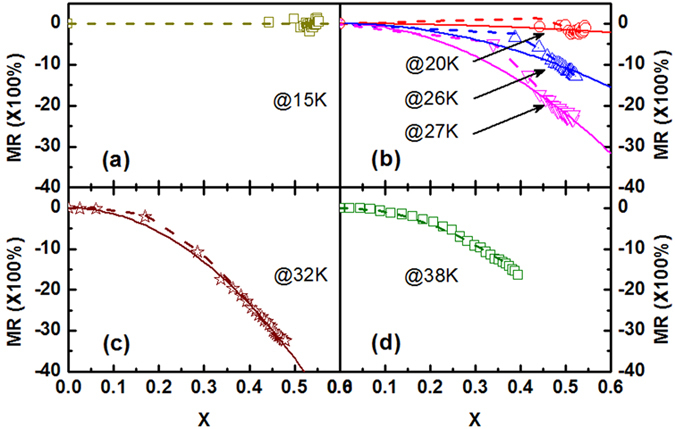
Comparisons between calculated and experimental results. Relationships between MR and X at T = 15 K, 20 K, 26 K, 27 K, 32 K and 38 K, respectively. X = M(H, T)/M_eff_. The scattered symbols are the experimental data. The dotted lines are the calculated results. The quadratic dependence of MR = *β*(T)X^2^ was plotted in solid lines.

The magnetic field dependence of MR at various temperatures has been measured and calculated^8^. Here the data were quoted and shown in Fig. 9. On one hand, MR(X) can be calculated according to eq. (16, 17, 18). On the other hand, the experimental results of MR(X) can be calculated from MR(*H*, *T*) and M(*H*, *T*). The curves for X dependence of MR at *T* = 15 K, 20 K, 26 K, 27 K, 32 K and 38 K were plotted in Fig. 10. The dotted lines are the calculated results and the scattered symbols are the experimental results. The experimental results are in good accordance with the calculated results in all three temperature zones mentioned in eq. (16, 17, 18). This indicates that the assumption about *ρ*_M_ expressed in eq. (4) and the calculation of MR are reasonable. And also we can see that MR is indeed closely related magnetic structure, magnetic transition and magnetizing process in PrGa compound.

Many works on MR in granular system have been reported, and it indicates that MR and X have the quadratic relationship MR = −*β*(*T*)X^2^ in granular materials^9^,^26^,^27^. So it is necessary to discuss whether the quadratic relationship is still appropriate in bulk PrGa compound. From eq. (16, 17, 18) and Fig. 10, it is clear that the MR for T < 17 K does not obey the quadratic relationship and the result for T ≥ 38 K matches the quadratic relationship strictly. The relationship of MR and X has more complex form in the temperature range between 17 K and 38 K according to eq. (17). The MR results at *T* = 20 K, 26 K, 27 K and 32 K shown in Fig. 10(b,c) are in this temperature range. To make it easy to compare, the function of MR = −*β*(*T*)X^2^ is also plotted in Fig. 10(b,c) in solid line. It shows that the experimental result for *T* = 32 K can be considered to obey the quadratic relationship approximately. However, the experimental results for *T* = 20 K, 26 K and 27 K show considerable deviations from the MR = −*β*(*T*)X^2^ relation. In order to find out why the approximation is effective in Fig. 10(c) and why the approximation is ineffective in Fig. 10(b), we need to review the derivation formula of MR in the temperature range between 17 K and 38 K. According to the second part of eq. (17), if M_0_(*T*) can be approximated as zero, MR can be expressed as MR = −*β*(*T*)X^2^. The temperature dependence of M_0_(*T*) is shown in Fig. 8. It shows that M_0_(20 K), M_0_(26 K) and M_0_(27 K) are 1.7 *μ*_B_, 1.3 *μ*_B_ and 1.0 *μ*_B_, respectively, while M_0_(32 K) is only 0.5 *μ*_B_ which is relatively smaller than each of the above three. As an approximation, we treat M_0_(32 K) as zero, and then obtain the quadratic relationship in the second part of eq. (17). Further, if the X_cr_ at 32 K is neglected, MR 32 K can be expressed as MR = −*β*(*T*)X^2^ approximately. However, M_0_(T) at 20 K, 26 K and 27 K is relatively large and it cannot be approximated as zero. Therefore, MR curves deviate noticeably from the quadratic relationship shown in Fig. 10(b).

The experimental results of MR showed a good agreement with the calculations. Further analysis indicates that the relationship between MR and X can be expressed as MR = −*β(T*)X^2^ in approximation only if the ordered magnetic moment at zero field is small enough. According to our systematic study on the magnetic structure, magnetic transition, MCE and MR, we obtained a more comprehensive understanding on the magnetic field controlled mechanism of the PrGa compound. This work will provide useful guidance for exploring new materials with good performance.

## Methods

### Sample fabrication and physical property measurements

Polycrystalline PrGa and YGa compounds were prepared by arc melting Pr/Y and Ga elements in Argon atmosphere and the molten salts were rotated several times to ensure the homogeneity. The annealing and quenching procedure are carried out afterwards. The purity of the starting elements is more than 99.9%. Crystal structure of YGa was determined by powder X-ray diffraction (XRD). Crystal structure and magnetic structures of PrGa at different temperatures were determined by high-resolution powder neutron diffraction experiment. Thermal magnetization and isothermal magnetization curves were measured on the Quantum-designed Vibrating Sample Magnetometer (VSM). Heat capacity data and resistance data were obtained from the Physical Property Measurement System (PPMS).

### Neutron powder diffraction experiments

High-resolution neutron powder diffraction data were collected using the BT1 32-detector diffractometer at the NIST Center for Neutron Research (NCNR). A Cu (311) monochromator with the wavelength λ = 1.5403(2) Å and in-pile collimation of 60’ were used. Data were collected over the 2-theta range of 3–168° with a step size of 0.05°. A closed cycle refrigerator (CCR) was used in temperature dependent measurements ranging from 4.5 K to 295 K. The Rietveld refinements were performed using GSAS program.

## Additional Information

**How to cite this article**: Zheng, X. Q. *et al.* The physical mechanism of magnetic field controlled magnetocaloric effect and magnetoresistance in bulk PrGa compound. *Sci. Rep.* **5**, 14970; doi: 10.1038/srep14970 (2015).

## Figures and Tables

**Table 1:** Refined structural parameters of PrGa compound.

Atoms	Parameters	4.5 K	15 K	20 K	25 K	28 K	30 K	33 K	36 K	40 K	295 K
	*a* (Å)	4.4545(3)	4.4545(3)	4.4545(3)	4.4538(3)	4.4533(3)	4.4522(3)	4.4515(4)	4.4506(3)	4.4504(4)	4.4494(4)
	*b* (Å)	11.2533(9)	11.2530(9)	11.2527(9)	11.2547(9)	11.259(1)	11.261(1)	11.264(1)	11.265(1)	11.267(1)	11.312(1)
	*c* (Å)	4.1878(3)	4.1880(3)	4.1877(3)	4.1875(3)	4.1865(3)	4.1860(3)	4.1861(3)	4.1862(3)	4.1857(3)	4.1953(4)
	*V* (Å^3^)	209.93(4)	209.93(4)	209.91(4)	209.90(4)	209.91(4)	209.87(4)	209.90(5)	209.88(4)	209.89(5)	211.15(5)
Pr	*y*	0.1416(2)	0.1419(2)	0.1418(2)	0.1421(2)	0.1420(2)	0.1425(3)	0.1424(3)	0.1426(3)	0.1429(3)	0.1441(3)
	*M*_*x*_*/M*_*y*_ (*μ*_B_)	0	0	0	0	0	0	0	0	0	0
	*M*_*z*_*/M* (*μ*_B_)	2.56(4)	2.43(4)	2.22(4)	1.90(4)	1.05(6)	0.6(1)	0.6(1)	0.65(9)	0	0
	*ϕ/*θ(deg.)	0	0	0	0	0	0	0	0	0	0
Ga	*y*	0.4301(2)	0.4301(2)	0.4305(2)	0.4302(2)	0.4303(2)	0.4304(2)	0.4306(2)	0.4304(2)	0.4306(2)	0.4304(2)
	*Rp* (%)	6.15	6.01	6.08	5.95	6.08	5.77	6.01	6.07	6.12	5.85
	*wRp* (%)	7.49	7.26	7.35	7.24	7.44	7.09	7.39	7.29	7.29	7.08
	χ^2^	1.363	1.274	1.300	1.281	1.292	1.276	1.313	1.224	1.246	1.458

Space group *Cmcm*, Atomic positions: Pr (0, y, 0.25); Ga (0, y, 0.25). a, b, c and V are lattice constant and volume of unit cell. M, M_x_, M_y_ and M_z_ are the total ordered magnetic moment and the individual component of moment along a, b and c axis, respectively. *ϕ* and *θ* are the angle between the ordered magnetic moment and z axis.
